# The Search for a Universal Treatment for Defined and Mixed Pathology Neurodegenerative Diseases

**DOI:** 10.3390/ijms252413424

**Published:** 2024-12-14

**Authors:** Danton H. O’Day

**Affiliations:** 1 Department of Biology, University of Toronto Mississauga, Mississauga, ON L5L 1C6, Canada; danton.oday@utoronto.ca; 2 Cell and Systems Biology, University of Toronto, Toronto, ON M5S 3G5, Canada

**Keywords:** neurodegeneration, mixed pathology, calcium dysregulation, calmodulin, transglutaminase 2, toxic protein aggregation, proteinopathies

## Abstract

The predominant neurodegenerative diseases, Alzheimer’s disease, Parkinson’s disease, dementia with Lewy Bodies, Huntington’s disease, amyotrophic lateral sclerosis, and frontotemporal dementia, are rarely pure diseases but, instead, show a diversity of mixed pathologies. At some level, all of them share a combination of one or more different toxic biomarker proteins: amyloid beta (Aβ), phosphorylated Tau (pTau), alpha-synuclein (αSyn), mutant huntingtin (mHtt), fused in sarcoma, superoxide dismutase 1, and TAR DNA-binding protein 43. These toxic proteins share some common attributes, making them potentially universal and simultaneous targets for therapeutic intervention. First, they all form toxic aggregates prior to taking on their final forms as contributors to plaques, neurofibrillary tangles, Lewy bodies, and other protein deposits. Second, the primary enzyme that directs their aggregation is transglutaminase 2 (TGM2), a brain-localized enzyme involved in neurodegeneration. Third, TGM2 binds to calmodulin, a regulatory event that can increase the activity of this enzyme threefold. Fourth, the most common mixed pathology toxic biomarkers (Aβ, pTau, αSyn, nHtt) also bind calmodulin, which can affect their ability to aggregate. This review examines the potential therapeutic routes opened up by this knowledge. The end goal reveals multiple opportunities that are immediately available for universal therapeutic treatment of the most devastating neurodegenerative diseases facing humankind.

## 1. Introduction

As the world population ages and as age-linked neurodegenerative diseases continue to increase, the growing financial, societal, and personal costs are immeasurable [1,2,3,4,5]. Neurodegenerative diseases (NDDs) such as Alzheimer’s disease (AD), Lewy body dementia (LBD), Frontotemporal dementia (FTD), and Huntington’s disease (HD) are primarily associated with a loss of cognitive functions that not only impact different types of learning and memory but also, over time and depending on the brain regions affected, socialization, personal awareness, and physical mobility [6]. Usually, these diseases progress from the loss of basic cognition to dementia and, ultimately, death. Other NDDs, such as amyotrophic lateral sclerosis (ALS) and Parkinson’s disease (PD), predominantly impact motor activity, causing uncontrollable shaking, loss of balance, and poor coordination [6]. Each neurodegenerative disease is characterized by toxic biomarkers that impact specific brain regions to produce associated symptomology. However, most NDDs are not pure but instead show crossover characteristics, called mixed pathologies, associated with other degenerative diseases [7,8].

Seven toxic biomarkers predominate the major NDDs: amyloid beta (Aβ), phosphorylated Tau (pTau), alpha-synuclein (αSyn), mutant huntingtin (mHtt), fused in sarcoma (FUS), superoxide dismutase 1 (SOD1), and TAR DNA-binding protein 43 (TDP-43) [6,9,10,11,12,13,14,15,16,17,18,19]. Here, we will examine those classic toxic biomarkers that have been used to define AD (Aβ, pTau), ALS (pTau, TDP-43, FUS, SOD1), FTD (pTau, TDP-43, FUS, SOD1), HD (mHtt), and PD (αSyn), before covering their importance in mixed pathologies associated with each NDD. The aggregation of these toxic biomarkers is then examined in a search for common regulatory factors. One of those factors, calcium-regulated transglutaminase 2 (TGM2). is overviewed, followed by the significance of calcium dyshomeostasis in NDDs. Finally, the function of calmodulin (CaM) as a primary effector in calcium signaling, as a regulator of TGM2, and as a binding factor for specific toxic biomarkers will reveal potential common targets for developing a potentially universal therapeutic treatment.

## 2. Specific Toxic Biomarkers Characterize the Major Neurodegenerative Diseases

Protein aggregates lie at the heart of most, if not all, neurodegenerative diseases. The general term for these protein aggregate-based NDDs is proteinopathies [20,21]. The most common proteins involved in proteinopathies, as revealed by disease-specific and age-dependent studies, include Aβ, Tau, mHtt, αSyn, SOD1, TDP-43, and FUS, as introduced above and detailed below. Some toxic proteins are aggregation-prone (e.g., prion protein, PrP; TDP-43; FUS), being able to spontaneously aggregate to overcome the intrinsic disorder, while others (e.g., Aβ, Tau, mHtt, αSyn, SOD1) have been shown to require enzymatic intervention involving TGM2 [22,23,24,25,26]. While many proteinopathies share aggregates of these same proteins, the resulting pathological effects depend upon the brain locations where those proteins accumulate. The aggregation and neurological damage caused by PrP have been well-reviewed and are not relevant to the focus of this article [27,28].

*Alzheimer’s Disease (AD).* The age-linked neurodegenerative disease AD is the major cause of dementia internationally, causing its sufferers to experience a progressive loss of memory, impaired communication, depression, and other behavioral issues [29,30]. As first revealed by Alois Alzheimer, traditionally, AD is identified by two primary biomarkers, extracellular Aβ plaques and intracellular pTau-based neurofibrillary tangles (NFTs) [9,31]. Plaques are generated from Aβ peptides that typically vary in size from 38 to 42 amino acids. Aβ oligomerizes, aggregates, and associates with other cellular constituents to produce plaques of various types. It is widely considered that Aβ42 oligomers are the primary toxic element during amyloidogenesis. Tau, encoded by the *MAPT* gene, binds to and functions in the assembly and stability of microtubules [32]. Phosphorylation of Tau (pTau) causes its release from microtubules, allowing it to form aggregates that develop into NFTs inside neurons. A second classic biomarker for AD, pTau, is also a common proteinopathy in other NDDs. Proteinopathies involving Tau are called tauopathies. Aβ and Tau have multiple neurotoxic effects, some of which are collaborative. They can exacerbate early calcium dysregulation, disrupt mitochondrial functions, and cause neuroinflammation, among other events [10,33].

*Lewy Body Dementia (LBD).* LBD, which also goes by the name dementia with Lewy bodies (DLBs), is a progressive disease characterized by the presence of accumulations of the toxic biomarker protein αSyn that localize in intracellular deposits called Lewy bodies (LBs) [34,35]. αSyn oligomers, the most common form of the biomarker, cause synaptic dysfunction and neuron death. LBs are roughly spherical cytoplasmic inclusions that are rich in αSyn but also include other proteins such as alpha B crystallin, neurofilament protein, and ubiquitin [36]. LBs are associated with the neurological problems of LBD, including changes in behavior, mood, movement, and the ability to think clearly, among others, such as depression, sleep problems, and hallucinations. As one of the most common NDDs after AD, LBD is difficult to diagnose because its symptoms overlap with those seen in other psychiatric disorders [35].

*Parkinson’s Disease (PD).* Parkinson’s Disease (PD) is a slow, progressive motor disease characterized by shaking and stiffness along with the loss of balance and coordination, plus mild cognitive impairment that will progress to dementia [19,37,38]. PD is not only the second most common NDD overall, but the number of individuals suffering from the disease is increasing faster than any other neurodegenerative disease. Increasing in frequency after age 65, PD sufferers exhibit a loss of dopaminergic (DA) neurons and decreased dopamine levels in the substantia nigra pars compacta that are responsible for its primary motor symptomology. Intraneuronal αSyn rich LBs characterize PD [39]. PD differs from other types of LBD based on the localization of LBs in those neurons. LBs can also contain parkin along with neurofilaments and ubiquitin plus other cellular constituents, depending on the type of LBs and their location. Encoded by the SNCA gene, αSyn’s significance in PD pathogenesis has been associated with its aggregation into toxic oligomeric protofibrils, an event mediated by the NAC (non-Aβ component; amino acids 61–95) domain [39,40].

*Frontotemporal Dementia (FTD)*. Originally called Picks disease, FTD is the second most common cause of early onset dementia and the third main cause of dementia in general, resulting in major changes in abstract thinking, behavior, executive thinking, and language [41]. FTD encompasses several brain diseases that, as its name implies, predominantly impact the frontal and temporal lobes of the brain that oversee behavior, language, and personality [41,42,43]. Atrophy of different lobe regions generates the resulting pathophysiology. FTD is associated with between 10 and 20% of dementia cases. The proteins TDP-43, FUS, and SOD1 have been implicated as risk factors for the disease [44,45,46]. FTD is also referred to as frontotemporal lobar degeneration (FTLD), but FTLD comprises a larger group of NDDs, of which FTD is a subgroup. The primary differences between them are the parts of the brain that are affected and, as a result, the pathophysiological results of those localizations. Relevant to this review, FTD shares overlap with ALS related to tau, TDP-43, and FUS [47]. Mislocalization and aggregation of TDP-43 is a biomarker for both NDDs [15]. In a mouse model of ALS, TDP-43 is known to mis-localize and aggregate, forming toxic fibrils under pathological conditions [48]. Coupled with other work, it remains in question whether the mislocalization of TDP-43 from the nucleus to the cytoplasm has more impact than protein aggregation in symptomology, though it is more likely they each have specific negative impacts [49,50].

*Amyotrophic lateral sclerosis (ALS).* As a motor neuron disease, ALS affects the control of muscles (arms, legs) and breathing, ultimately causing death [51,52,53,54]. While uncurable, it does not significantly impact intelligence, hearing, seeing, or thinking. While familial and sporadic forms of ALS are clinically and pathophysiologically indistinguishable, mutations in over one dozen genes, especially *C9ORF72*, *SOD1*, *FUS*, and *TARDBP*, underlie familial ALS. More than 200 different mutations in the SOD1 gene are responsible for up to two percent of sporadic ALS and as much as one-fifth of familial cases [2,14]. Primarily localizing to the cytosol and mitochondria, SOD1 is a copper- and zinc-binding antioxidant enzyme that converts superoxide radicals to hydrogen peroxide and oxygen [14,55]. Its role in disease pathology is still under analysis. Aggregates of mutated SOD1, FUS, and TDP-43 are present in LB-like hyaline inclusions found in ALS motor neurons [14,56]. These proteins are structurally and functionally unrelated, so understanding what drives their aggregation as a step in the formation of ALS Lewy-body-like inclusions is under analysis and may be different for each inclusion protein [57,58]. For example, aggregation of TDP-43 in ALS appears to depend on the type of mutation, post-translational modifications, and other factors [13].

ALS shows significant overlapping attributes clinically, genetically, and morphologically with FTD [59]. While ALS primarily affects movement, FTD impacts cognition. The two share common gene mutations (e.g., *C9ORF72*, *SOD1*, *FUS*, and *TARDBP*), but it is where these genes are expressed that leads to the symptomology of each disease. Since they are known to form disease-related aggregates, SOD1, FUS, and TDP-43 (encoded by *TARDBP*) are relevant to this review. TDP-43, FUS, and SOD1 can be organized in several different types of inclusions with different cellular locations, but neuronal toxicity is not a product of a specific type of aggregate or inclusion [60].

*Huntington’s disease (HD).* HD is an age-dependent, hereditary, progressive, uncurable, and ultimately fatal disorder with symptoms that include, but are not limited to, cognitive issues, uncontrollable muscle activity, and psychological problems [61,62]. Mutations in the *HTT* gene on chromosome 4 are responsible for this NDD, which primarily causes the degeneration and death of striatal medium-sized spiny neurons (MSNs) and cortical pyramidal neurons [63]. Encoded by the *HTT* gene, normal huntingtin (Htt) protein possesses an N-term polyglutamine (poly Q) repeat of 9-35 glutamines encoded by triplet CAG repeats [64]. Mutated Htt (mHtt) has a more extensive, more toxic poly Q repeat that is longer than 35 glutamines, with the resulting pathophysiology directly related to the increase in poly Q length. A recent study using expansion microscopy provided evidence that the polyglutamine aggregates of HD disrupt nuclear envelope integrity and impede its repair [65].

## 3. Mixed Pathologies: Major NDDs Share Toxic Biomarkers

The co-existence of neuropathological protein hallmarks for more than one NDD in a single individual exemplifies a mixed NDD pathology [7]. Mixed pathologies are also referred to as coexisting pathologies, concomitant pathologies, co-pathologies, mixed proteinopathies, and other names [6,66]. Multiple neurodegenerative diseases show pathological overlap with different combinations of proteinopathies and age-related pathologies, amongst others [26]. Since patients are often found with mixed pathologies, most NDDs are currently viewed by experts as multifaceted diseases expressing diverse symptoms, disease progression, and therapeutic responses [7,8,67,68]. Diagnosed NDDs are rarely “pure” because they are often coupled with other neuropathologies as well as age-related ailments, leading to a high level of disease heterogeneity [8]. For example, AD sufferers commonly show Lewy body pathology or TDP-43 inclusions, among others [8,69]. Dementia has been reported to be more likely with multiple proteinopathies, as exemplified in one study where NFTs, Aβ plaques, TDP-43 inclusions, and α-synuclein-associated Lewy pathology were all found in a single patient [70]. Mixed pathologies increase the chances of cognitive impairment and dementia.

**Figure 1:**
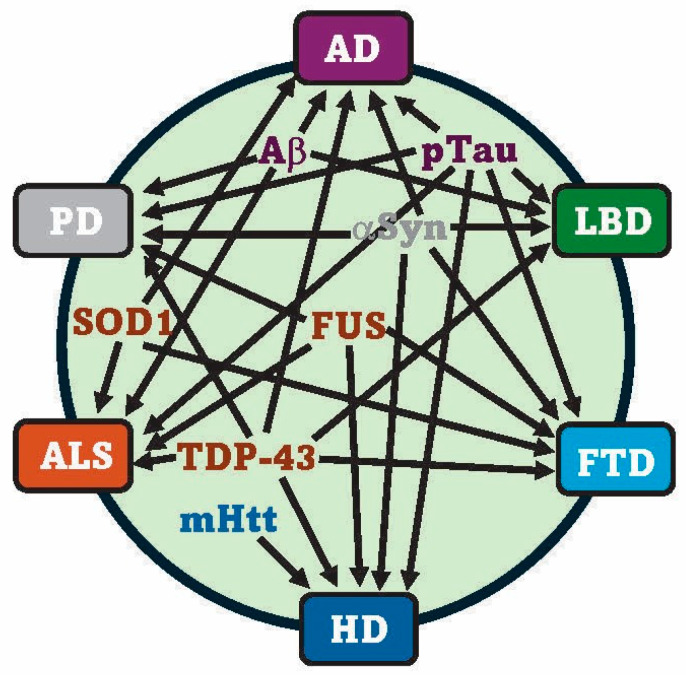
Toxic protein biomarkers shared by specific and mixed pathology NDDs. The references supporting these relationships are cited in the main body of the text. The color of the individual toxic biomarkers indicates the primary NDD with which they are associated. *Abbreviations*: amyloid beta (Aβ), phosphorylated Tau (pTau), alpha-synuclein (αSyn), mutant huntingtin (mHtt), fused in sarcoma (FUS), superoxide dismutase 1 (SOD1) and TAR DNA-binding protein 43 (TDP-43).

Mixed NDD pathologies are likely due to the complex interplay of environmental factors, lifestyle, genetic mutations, toxic biomarkers, and cellular pathways (e.g., neuroinflammation) [66]. Mixed pathologies are age-related, and so older patients suspected of having a specific NDD will often express age-dependent co-pathologies [8]. For example, by 80 years of age, pTau NFTs are present in 95–99% of individuals, Aβ plaques in 60–70%, TDP-43 inclusions in 30–40%, and Lewy pathologies in 15–30%. Mixed pathologies can confuse disease diagnosis, an effect that may not become clear until postmortem analysis [71,72]. They can also exacerbate symptoms and increase the rate of disease progression. Is it possible that mixed pathologies are the norm, not the exception? If so, this points to the value of developing therapies that are universal versus disease- or biomarker-specific. Figure 1 summarizes the shared biomarkers between defined and mixed NDDs.

*AD*. As the most common NDD, AD demonstrates various mixed pathologies. Vascular dementia is the most common form of mixed AD dementia [1]. Around 50% of AD patients are found to also have αSyn-rich LBs, a mixed pathology known as AD with LBD [34,73]. With AD, age-related comorbid pathologies are frequently detected, including TDP-43 (15–35% of early age cases vs. 50% late) and αSyn (~25% vs. 50%) [8]. Such co-pathologies have a significant impact on the results of clinical trials. SOD1-linked pathology has also been found in AD patients, where it is intimately linked to oxidative damage with Aβ directly interacting with the enzyme, impairing its enzymatic activity [12].

*LBD*. LBD is a progressive degenerative brain disorder that shares features with other NDDs, including DLB and PD, resulting in attributes of Parkinsonism and psychosis [74]. In addition to αSyn associated with LBs, LBD often shares characteristics of AD, expressing Aβ, amyloid plaques, and pTau-generated NFTs, often leading to the misdiagnosis of the disease. TDP-43 has been detected in up to 63% of autopsy-confirmed LBD cases [71].

*PD*. Though primarily recognized as a motor disease, PD also expresses non-motor symptomology. It has been estimated that more than 90% of PD cases involve mixed pathologies [75,76]. While αSyn-rich LBs and Lewy neurites are consistently observed in the nigrostriatal system, other toxic biomarkers are common. Thus, Aβ, pTau, αSyn, and TDP-43 deposits have been detected in LBs in PD, LBD, and other synucleinopathies [66]. Aβ plaques and NFTs occur in 10% of PD cases, a percentage that increases to as much as 70% in PD dementia with LBs, while TDP-43 has been uncovered in up to 30% of patients [76]. FUS has also been identified in persons suffering from PD [31]. The inter-relationship and interactions between αSyn and other biomarkers and their implications for disease onset and progression are complex and still under analysis [77].

*ALS*. A progressive, neurodegenerative disease, ALS, commonly called Lou Gehrig’s disease, is the primary motor neuron disease with sporadic and familial forms. The disease is clinically heterogeneous, with over 120 genes linked to its onset and progression, but with the expression of variants of the proteins TDP-43, FUS, C90RF72z, and SOD1 being predominant [78,79]. The impact of each of these genes and the impact of environmental factors in sporadic and familial ALS have been well-reviewed [80,81]. Both Aβ and pTau have been found in ALS sufferers [33].

*FTD*. Neurodegeneration in the frontal and anterior temporal lobes characterizes FTD [43]. Primarily sporadic, as much as 40% of FTD cases have a genetic influence primarily involving C9orf72, TDP43, and FUS [42]. The disease is characterized by protein aggregates rich, to varying degrees, in TDP-43, Tau, and FUS [82]. A SOD1 mutation has been associated with FTD and ALS [83]. In 2015, a case of frontotemporal lobar degeneration with αSyn was published [84].

*HD*. St-Amour and colleagues “observed that 88% of HD patients with Vonsattel grade 4 neuropathology displayed at least one non-Htt proteinopathy compared to 29% in controls” [20]. Commonly, the aggregation of TDP-43 and pTau is associated with HD progression. The presence of pTau in the brains of HD patients was first recognized over 40 years ago, and continued research has revealed the presence of different Tau isoforms in early and late-onset HD, with a total of around 60% of patients showing Tau pathology [85]. The presence of αSyn aggregates has also been revealed within mHtt inclusions [86]. In addition, FUS has also been detected in HD patients [25]. Evidence exists for amyloid inclusions in HD patients with classic and mixed pathologies, but the evidence for Aβ -based inclusions has not been established [87,88].

## 4. Calcium Dysregulation and Calmodulin Function in Neurodegeneration

**Figure 2:**
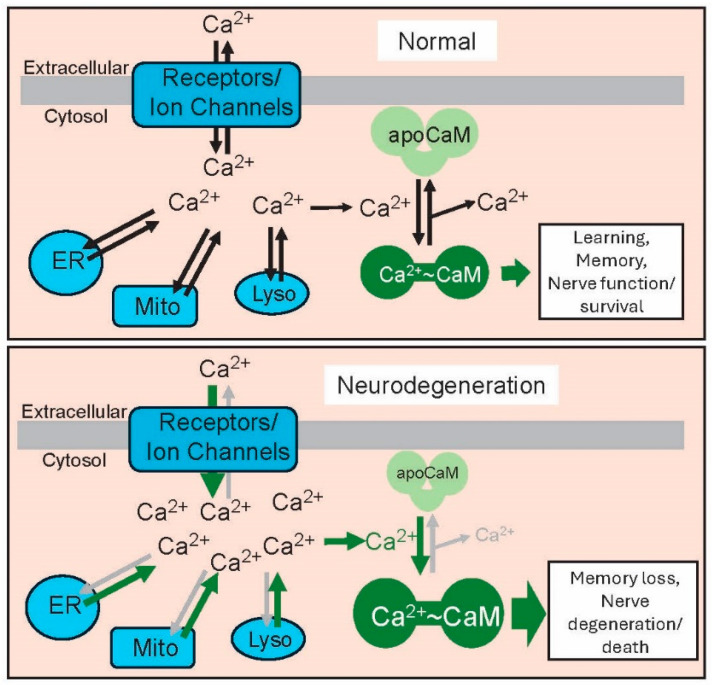
Calcium–calmodulin mediated signal transduction in normal neurons (**top panel**) and neurodegeneration (**lower panel**). This essential signaling pathway mediates learning and memory as well as the essential functions and survival of brain cells. During neurodegeneration, the unregulated influx of calcium ions mediated by various receptors and ion channels increases cytosolic calcium to toxic levels, in turn increasing the level of Ca^2+^-CaM, causing changes implicated in memory loss, nerve degeneration, and death. Abbreviations: ER, endoplasmic reticulum; Mito, mitochondria; Lyso, lysosomes.

Despite the apparent differences between the diverse array of neurodegenerative diseases, there is an early and critical event common to most, if not all, of them: the dysregulation of calcium levels (Figure 2). This dysregulation, an early unregulated and damaging increase in cytosolic levels of calcium ions, was first recognized in AD as the Calcium Hypothesis but subsequently became recognized as a common feature of multiple NDDs [89,90,91,92]. Among other events, neuronal Ca^2+^ signaling mediates learning and memory, neurogenesis, neurotransmitter synthesis and release, membrane excitability, and energy metabolism [93]. The level of the divalent cation is precisely regulated (10^−7^ to 10^−8^ mol) such that small and persistent disruptions can be harmful [90,91]. In healthy neurons, intracellular calcium levels are maintained through the intake and release of calcium across the cell membrane via the action of receptors and ion channels and from major intracellular stores contained in the endoplasmic reticulum, mitochondria, and lysosomes. Calcium can bind to apo-CaM to produce Ca^2+^-CaM.

The Ca^2+^-binding protein CaM is a primary and critical Ca^2+^ sensor and effector in the brain, where it binds to essential proteins involved in synaptic functions, learning, and memory and is linked to multiple NDDs [94,95,96]. In its calcium-free apoCaM configuration, this small calcium sensor and effector binds to and regulates a comparatively small number of CaM-binding proteins (CaMBPs). However, when activated by calcium, Ca^2+^-CaM can bind to and regulate hundreds of proteins, including multiple calcium ion channels and receptors that oversee the intracellular regulation of calcium levels. The increased calcium levels in NDDs are due to the altered influx and efflux across the cell membrane and from intracellular stores, potentially leading to the downstream over-production of Ca^2+^-CaM and causing memory loss, neurodegeneration, and neuron death, as discussed for each NDD below. Here, a short summary of evidence supporting the early role of calcium dyshomeostasis is presented, along with its impact on Ca^2+^-CaM signaling.

*AD*. Calcium dysregulation plays an early and continuous role in AD, where it is involved in autophagy, mitochondrial disruption, and neuroinflammation [91,92,96,97,98]. Calcium dysregulation in AD impacts intracellular signal transduction and mitochondrial calcium overload, in turn resulting in reduced ATP generation and increased ROS [97,98]. Increased unregulated calcium increases the synthesis and aggregation of Aβ and Tau phosphorylation, events that feed back to further augment altered calcium levels. Aβ oligomers not only generate unregulated calcium-permeable pores in the cell membrane but also bind to and activate numerous calcium ion channels. Multiple critical putative CaMBPs regulate amyloidogenesis and neurofibrillary tangle formation, leading to the Calmodulin Hypothesis of AD [99]. For example, amyloid beta protein precursor (AβPP), β-secretase (BACE1), presenilin 1 (PSEN-1), and Aβ are all CaMBPs [100]. In addition, most of the key receptors and ion channels involved in calcium levels that are regulated by CaM are also central to calcium dysregulation in multiple NDDs, including AD, HD, and PD [101]. In different ways, Aβ, Tau, and αSyn each disrupt calcium signaling, suggesting that their combined effects might be responsible for the more rapid neurodegeneration seen in AD sufferers with mixed pathology [8].

*HD*. Calcium dysregulation is a central and early event in HD, an event that is also critical for TGM2 activation [102,103,104]. Together, they drive the formation and aggregation of Htt. In addition to initiating some of the critical symptomology of the disease, the classical marker mHtt also binds to IP3Rs, leading to an additional increase in calcium release from the ER [102,105]. As with AD, increased levels of cytosolic calcium disrupt mitochondrial function, decreasing metabolism (ATP production decreases) and increasing ROS. Two downstream targets of the calcium increase, calpain, and calmodulin, are involved in HD. The calcium-dependent protease calpain cleaves mHtt, generating polyQ fragments that form toxic aggregates that transform into inclusions [106].

*PD*. The dysregulation of intracellular calcium levels is linked to PD symptomology and, in keeping with this, CaM is also involved in the early events of the disease [107]. Calcium dysregulation is an early event partly due to the increased levels of Cav1.3 voltage-gated calcium channels in substantia nigra neurons [108,109]. The increased cytosolic calcium levels, coupled with the mitochondrial disruption of ATP production and increased reactive oxygen species (ROS), cause an increase in αSyn aggregates that, in turn, feed back to augment calcium levels by binding to voltage-gated calcium channels and other channels and receptors [110,111,112]. Pesticides that are implicated in the onset of PD and other NDDs have also been shown to initiate toxic cytosolic increases in intracellular calcium levels, leading to neuronal dysfunction [113]. There is a great diversity in pesticide types, their chemical structures, environmental stability, biotoxicity, and potential for human exposure. Despite an evaluation of a diversity of insecticides, fungicides, fumigants, herbicides, and rodenticides, among others, defining the specific types of pesticides that could cause PD is challenging. That said, ten neurotoxic pesticides have gained attention, including certain fungicides (copper sulfate and folpet), herbicides (diquat, endothall, and trifluralin), and insecticides (dicofol, endosulfan, naled, and propargite) [114]. In keeping with the importance of calcium dysregulation, a large number of CaMBPs involved in calcium and CaM-mediated signal transduction components are expressed in PD, including receptors (NMDAR, AchR, and Adenosine A2AR), biomarkers (αSyn, Aβ, Tau, and D2DR), and enzymes (CaMKII, PP2B, and cdk5) [115,116].

*LBD*. Calcium dysregulation is a critical early event in LBD because it is involved in ER stress, mitochondrial malfunction, and other critical pathways driving the degeneration of disease-specific subcortical neurons, cortical neurons, and dopaminergic neurons in the substantia nigra pars compacta (SNc) [117,118]. It also initiates αSyn expression and oligomerization, events that further enhance the toxic increase in calcium levels associated with this NDD [119]. The increased calcium binds to downstream CaBPs, including members of the CaM superfamily (e.g., calcium binding protein1 and CaM), in turn further augmenting calcium levels and LBD pathology [118].

*ALS*. Calcium homeostasis is altered in ALS, and increased cytosolic calcium levels coupled with low levels of the calcium-binding proteins (e.g., parvalbumin and calbindin-D28K) contribute to cytotoxicity resulting in motor neuron degeneration and death [120,121,122,123]. Disrupted calcium homeostasis in ALS motor neurons results in increased cytosolic levels, an event exacerbated by mutated SOD1 [124,125]. In vitro studies on a motor neuron cell line demonstrated that increased cytosolic calcium levels led to increased neuronal NOS levels coupled with increased mutant SOD1 aggregation [126]. A bioinformatic analysis employing Ingenuity Pathway Analysis (IPA), STRING, and Cytoscape to analyze SOD1 and its link to ALS supports a role for CaM in SOD1-triggered neurodegeneration in ALS [14].

*FTD*. The importance of calcium dysregulation seen in the NDDs already covered has also been revealed for FTD [127,128]. The literature on the function of CaM in FTD is also extremely limited. However, evidence reveals that various CaMBPs, common to several other NDDs, are also involved in FTD neuroinflammatory events [96]. More to the point, as detailed below, various toxic biomarker proteins expressed in FTD and FTD with mixed pathologies are proven CaMBPs [116].

## 5. Transglutaminase 2: The Aggregation of NDD Toxic Biomarkers

Protein-glutamine gamma-glutamyltransferase 2 (TGM2, Uniprot P21980) is more simply known as transglutaminase 2. The ability of TGMs to cause protein polymerization by forming crosslinks (i.e., covalent isopeptide bonds) between glutamine and lysine residues in peptides coupled with transamination of primary amines to glutamines has led to a diversity of bioengineering and biotechnological applications [129]. These include but are not limited to improving food nutritive value, quality, and texture, biomedical applications (e.g., hydrogels for drug delivery and tissue bioengineering; DNA/protein conjugation), and advances in pharmaceutical development [129,130]. TGM2 is the most studied member of the multi-functional nine-member TGM family that includes TGM1-7, coagulation Factor XIIIa subunit (plasma TG), and erythrocyte membrane protein band 4.2 [131]. Enzymatically, TGM2 functions as a transamidase, GTPase, kinase, and disulfide isomerase. The enzymatic mechanisms for these catalytic events have been detailed [131,132,133,134]. Localized both intracellularly and extracellularly, TGM2 has been shown to be involved in cell growth, death, and differentiation, as well as fibrosis, inflammation, phagocytosis, and tissue repair [134]. As a result, altered TGM2 function has been linked to a number of human diseases, including celiac disease, diabetes, inflammatory disease, tissue fibrosis, various cancers, and, as detailed below, NDDs. Of relevance here, TGM2 is an enzyme that plays a critical and central role in the aggregation of multiple toxic biomarkers that characterize many primary and mixed NDDs.

**Figure 3:**
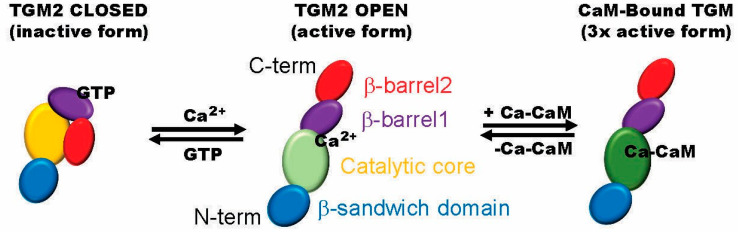
The regulation of transglutaminase 2 (TGM2). The enzyme is inactive when bound to GTP, but upon calcium binding, the protein unfolds, becoming active. The binding of CaM in the catalytic core is suggested. Calmodulin binding enhances TGM2 enzyme activity 3-fold. The details of these events are summarized in the main text. The color of the catalytic core area is suggestive of activity level (yellow, inactive; light green, active; dark green, enhanced activity). Figure after [131].

The cross-linking and transamidase activities of TGM2 are calcium-dependent events [129]. TGM2 has a GTP-bound closed conformation in unstressed normal cells that, in response to nucleotide removal and the presence of calcium, transforms it into a transamidase active open form (Figure 3) [135]. Thus, GTP is an inhibitor, while calcium is an activator of the transamidase activity of TGM2. Calcium-dependent TGM2 post-translationally cross-links and transaminates proteins. A cysteine residue in the active site of TGM2 interacts with g-caboxyamide groups in glutamine residues in the target protein, generating a g-glutamyl thioester that, in turn, reacts with nucleophilic amines to form a degradation-resistant amine or covalent isopeptide bond [136]. The result is an alteration of the protein’s conformation, which can lead to the production of insoluble supramolecular structures. These events underlie the formation of insoluble Aβ plaques, mHtt aggregates, and αSyn-rich Lewy bodies, the biomarkers for AD, HD, and PD, respectively. In contrast, in healthy cells, TGM2-mediated cross-linking is detectable but low due to insufficient calcium to fully activate it [137]. The early, unregulated increase in calcium levels generated in NDDs, as detailed above, causes the activation of TGM2, driving the cross-linking of toxic biomarkers. In keeping with this, TGM2 has been linked to multiple neurodegenerative diseases, including AD, ALS, HD, LBD, MS, and PD, among others [138,139,140,141]. The upregulation of TGM2 mRNA and protein is also observed in multiple NDDs, including AD and HD, along with other polyglutamine expansion diseases and PD [142].

*AD*. In AD brains, TGM2 is expressed at increased levels and accumulates in senile plaques and NFTs [143]. TGM2 crosslinks Aβ as well as pTau, generating Aβ peptides to form oligomers prior to plaque formation, and Tau dimers are involved in either the formation or stabilization of NFTs [144]. Increased calcium levels also cause an increase in TGM2 activity, driving the formation of Aβ oligomers [104,145,146]. TGM2 colocalizes with later stages of plaque formation but is likely via protein–protein binding rather than involvement in cross-linking reactions [147]. Immunochemical studies of human AD brains have revealed the colocalization of TGM2 with various stages in the formation of both NFTs and Aβ plaques in appropriate disease-linked brain locations [148].

*HD*. In HD, calcium dysregulation activates TGM2, resulting in the cross-linking of mHtt to produce mHtt oligomers. TGM2 colocalizes with mHtt in HD inclusions [149,150]. TGM2 enzyme activity levels are increased in the HD brain, primarily in the superior frontal cortex [151]. In HD neurons, CaM, HTT, and TGM2 colocalize [139]. The enzyme hydrolyzes the poly-Q repeat and also cross-links HTT fragments, forming insoluble deposits [139,143,150,152]. While calcium activates TGM2, activated CaM also binds to the enzyme, further increasing the cross-linking of mHtt. mHtt is also a CaMBP with a higher affinity for CaM binding than Htt [143,152]. Studies have shown that CaM antagonism decreases TGM2 activity, reducing mHtt cytotoxicity [153].

*PD and LBD*. The accumulation of αSyn in LBs and Lewy neurites, primarily in the substantia nigra, characterize PD and is associated with its symptomology. The activity of TGM2 is increased in PD patients [104]. Co-localization and immunoprecipitation studies reveal the association between TGM2 and αSyn in the substantia nigra where TGM2-dependent cross-linking of αSyn to generate oligomers has been revealed [110,154]. The overlap between PD and LBD is exemplified by the localization of TGM2 within LBs of both NDDs [143]. The enzyme phosphorylates αSyn Ser129, increasing its aggregation, an event correlated with disease severity [155]. It also catalyzes αSyn cross-linking, forming insoluble aggregates, the progenitors of LBs [143].

*ALS and FTD*. ALS and FTD not only share many attributes, but they also co-occur, which is not surprising since they share a number of critical genes that are expressed in different brain regions, generating disease-specific pathologies. In the early stages of ALS, serum TGM levels correlate with motor neuron degeneration and disease severity, indicating the enzyme is involved in disease pathogenesis [156]. TGM2 can accelerate neuroinflammation in ALS, likely contributing to the progressive selective neurodegeneration of disease-linked motor neurons in the brain and spinal cord [157]. As discussed above, while exhibiting different pathophysiologies, ALS and FTD share three common toxic proteins that are found in aggregates: TDP-43, FUS, and SOD1. Of these, the aggregation of TDP-43 and FUS has not been shown to involve TGM2. On the other hand, TGM2 not only binds to misfolded SOD1, but it is also linked to its oligomerization, events associated with disease pathogenesis, and neuroinflammation [51]. In transfected cells, TGM2 binds to mutated SOD1 (mSOD1), driving its calcium-dependent oligomerization [51]. Coupled with a concomitant increase in induced tumor necrosis factor-α, interleukin-1β, and nitric oxide in microglia indicated this TGM2-mediated aggregation also induces neuroinflammation. Cystamine, a TGM2 inhibitor, delays both oligomerization and microglial activation [141,158]. While TGM2 has been shown to regulate the aggregation of SOD1, how oligomerization occurs remains to be elucidated [51].

In summary, cross-linking of monomeric hallmark proteins (i.e., Aβ, pTau, mHtt, αSyn) generates toxic oligomers, the precursors of characteristic brain inclusions for AD (Aβ plaques, NFTs), HD (mHtt fibrils) and PD (Lewy bodies and neurites). TGM2 cross-links Aβ peptides to form oligomers; pTau is cross-linked to form anti-parallel dimers; mHtt is cross-linked to form insoluble fibrils; αSyn cross-linking generates fibrils. Thus, TGM2 is involved in the critical transition of hallmark biomarker proteins into the toxic oligomers, dimers, and fibrils that underlie the symptomology of those primary diseases.

## 6. TGM2 Is Regulated by Calmodulin

As covered above, the calcium dysregulation that characterizes multiple NDDs activates CaM, a primary downstream calcium-binding protein. As part of this initiated signaling, Ca^2+^-CaM binds TGM2, increasing its activity up to 3-fold [139,152,159]. Two CaMBDs were identified in human TGM2 (P21980) [160]. CaMBD1 (414KSINRSLIVGLKISTKSVGR433) contains three binding motifs (1-16, 1-12, 1-5-10), while CaMBD2 (665VVNFESDKLKAVKGFRNVII683) displays five motifs (two 1-12, 1-8-14, two 1-10). Antagonism of CaM with W5-hydrochloride in mHtt/TGM2 transfected culture cells inhibited mHtt aggregation by TGM2 [139,149]. Despite multiple reports verifying TGM2 as a CaMBP, this critical area of enzyme regulation remains to be studied.

## 7. Critical Toxic Biomarkers Bind to Calmodulin

*Aβ.* Aβ binds to Ca^2+^-CaM via the Aβ 25-35 neurotoxic region, and this binding inhibits Aβ fibrillization [161]. The CaMBD for Aβ42, which is shorter than typical binding domains, has been identified (25GSNKGAIIGLM35), revealing that it is a non-canonical CaMBD [161]. The high affinity binding for Ca^2+^-CaM is approximately 20 times higher than for apoCaM. With a K_d_ of 0.98 ± 0.11 nM, this indicates CaM is one of the primary targets for Aβ binding.

*Tau*. Ca^2+^-CaM binds to the second tubulin binding site repeat that includes a non-canonical CaMBD (bold letters in sequence: **V**TSKCGS**L**GN**I**HHKPGGG) not found in other CaMBPs [162,163]. Mandelkow and Mandelkow, 2012). Despite the revelation of CaM binding to Tau by multiple independent research groups, the significance of this binding remains to be clarified.

α*Syn*. While the binding of αSyn to CaM was verified years ago, the binding domain was not identified [164]. A recent search using the Calmodulin Target Database revealed a potential CaMBD (32KTKQGVAEAAGKTKEGVLYVGSKTKEGVVH50) containing two 1-12 binding motifs [160]. Martinez and colleagues demonstrated that Ca^2+^-CaM binding to αSyn enhances fibril formation [164].

*mHtt.* Human mHtt binds to CaM with greater affinity than Htt, indicating a role for polyQ tracts in the calcium-dependent binding [165]. (Bao et al., 1996). Zainelli and colleagues later revealed the direct relationship between mHtt, TGM2, and CaM in Huntington’s disease [139,149]. To summarize their detailed findings, mHtt, TGM2, and CaM co-immunoprecipitate and also colocalize to disease-specific inclusions in the nuclei of HD cortical neurons; TGM2 selectively cross-links mHtt with expanded poly Q over normal Htt; and, mHtt with expanded poly Q binds CaM with selective affinity over normal Htt [139,149]. A Calmodulin Target Database search revealed human Htt possesses two CaMBDs with multiple binding motifs (CaMBD1: (177NGAPRSLRAALWRFAELAHLVR197) containing three motifs (1-12, 1-8-14, 1-5-10); CaMBD2: (2535PLKALDTRFGRKLSIIRGIV2554) with five motifs (three 1-12, 1-8-14, 1-16)) emphasizing the ability of Htt to bind CaM [160].

*TDP-43, SOD1, and FUS*. Unlike other toxic biomarkers that bind to CaM, a literature search did not reveal any work performed on CaM-binding to SOD1, and a Calmodulin Target Database scan did not reveal any potential CaMBDs in the enzyme [160]. On the other hand, CaM function has been linked to SOD1-initiated neurodegeneration in ALS, but the mode of its involvement remains to be shown [14]. There appears to be no literature reports experimentally validating the binding of CaM to TDP-43 or FUS, but sequence scans for potential calcium-dependent, canonical CaMBDs revealed their existence in both of these proteins, suggestive of potential CaM binding [160]. FUS has a 19 aa putative CaMBD (301VADYFKQIGIIKTNKKTGQ319) with a single 1-5-10 binding motif. In contrast, a 19 aa CaMBD in TDP-43 (135VKKDLKTGHSKGFGFVRFT153) contains three binding motifs (1-16, 1-12, 1-14). While unlikely, this does not rule out the potential for non-canonical calcium-dependent CaM binding. No ALS-specific mutations fall within the presumptive CaMBDs of either FUS or TDP-43. It should also be noted that no IQ motifs characteristic of calcium-independent apoCaM-binding have been detected in FUS, TDP-43, or SOD1 [160].

## 8. Conclusions

**Figure 4:**
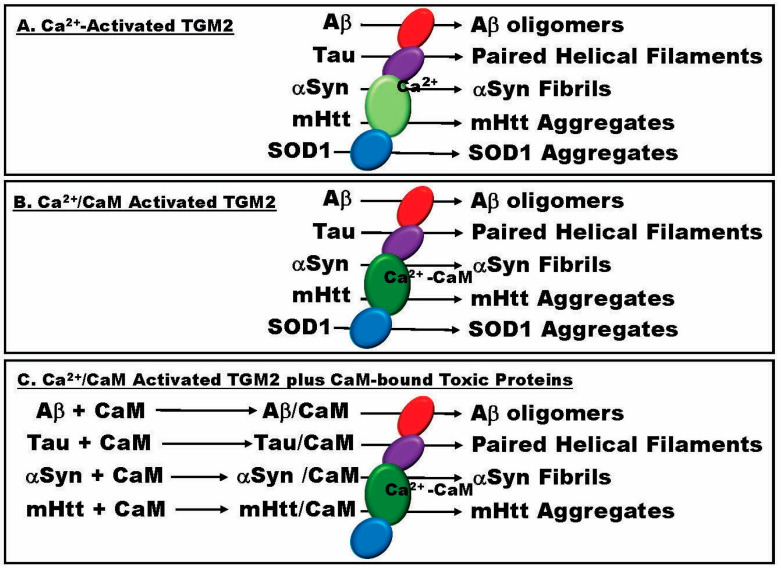
A diagrammatic representation of the potential interactions between calcium (Ca^2+^), calmodulin (CaM), and transglutaminase 2 (TGM2) and their potential implications for the aggregation of toxic biomarkers in neurodegenerative diseases. (**A**) Basic model for calcium-regulated TGM2 mediating toxic protein (Aβ, Tau, αSyn, mHtt, SOD1) aggregation. (**B**) Enhanced aggregation directed by Ca^2+^-CaM-bound TGM2. (**C**) The potential involvement of calmodulin binding to the aggregation of specific toxic proteins (Aβ, Tau, αSyn, mHtt). See text for details.

Supporting their comment with 18 references, Wilson and colleagues state that the aggregation of toxic hallmark proteins plays a central pathogenic role in neurodegenerative diseases, including but not limited to AD, ALS, FUS, FTD, HD, and PD [166]. Considering that TGM2 is the primary enzyme involved in the aggregation of toxic peptides linked to these NDDs, it follows that it should be considered a primary and almost universal therapeutic target for NDD treatments. As detailed here, research has identified six toxic proteins (Aβ, Tau, αSyn, FUS, SOD1, TDP-43) that are not only classic biomarkers for AD (Aβ, pTau), ALS (pTau, TDP-43, FUS, SOD1), FTD (pTau, TDP-43, FUS, SOD1), and PD (αSyn) but also contribute to mixed pathologies associated with each of those diseases as detailed above (Figure 4). In contrast, mHtt appears to be specific to HD alone. Each of these proteins forms toxic aggregates that are implicated in disease pathophysiology. The aggregation of Aβ, Tau, αSyn, SOD1, and mHtt is driven by TGM2, a calcium-dependent transamidase whose activity is activated by the increased calcium levels that characterize NDDs. The primary downstream target of calcium is CaM. Ca^2+^-CaM can bind to TGM2, increasing its activity up to 3-fold. The final piece of this complex regulatory puzzle is the ability of CaM to bind to and potentially affect the aggregation of Aβ, Tau, αSyn, and mHtt. Evidence suggests CaM may also bind TDP-43. Thus, CaM, as a result of its response to the calcium dyshomeostasis of NDDs, its ability to increase the activity of TGM2, and its binding to critical toxic biomarkers that are aggregated by TGM2, is a forefront protein that is central to both major pure and mixed pathology NDDs.

Because of the multifunctional activities of TGM2 and its involvement in multiple human diseases, it has been considered a primary therapeutic target, but developing specific inhibitors to use therapeutically has been a challenge [134,167]. While a number of specific inhibitors exist for the study of TGM2 in vitro, they are not ready for therapeutic use [153]. In the words of Yadav and Kim, “Numerous transglutaminase inhibitors exist and have enabled proof-of-concept investigations in disease models, but their poor pharmacokinetic/pharmacodynamic characteristics and/or lack of selectivity for the TG2 isoform have restricted their use in clinical applications” [168]. New inhibitors developed by Zedira GmbH are undergoing clinical trials for the treatment of celiac disease [131]. The direct targeting of TGM2 activity for the treatment of various neurodegenerative diseases has been reviewed [141]. Based upon their mode of action, three types of inhibitors have been generated: competitive amine inhibitors that bind to the active site to inhibit transamination, reversible inhibitors that reversibly block active site access, and irreversible inhibitors that form covalent linkages that inactivate the enzyme. [169,170]. Two amine inhibitors, cystamine and its reduced form, cysteamine, have each shown some positive results in the prevention of protein aggregation in in vivo models of HD and PD, but each suffers from either a lack of target specificity (cystamine) or undesirable side effects (cysteamine) [141,158,171]. The future development of modified forms of these inhibitors may be able to overcome these negative issues. While there are several reversible and irreversible inhibitors of TGM2, they have only been tested as therapeutic options for the treatment of various cancers [172]. To date, their ability to prevent the aggregation of toxic neurodegenerative proteins has apparently not been studied. Since the current approach to targeting TGM2 has met with little success, it might be time to look at CaM-binding to develop an effective therapy.

While TGM2 can be inhibited directly, the inhibition of CaM binding to TGM2 could prevent or reduce the early cross-linking of toxic peptides. Since TGM2 is normally active at low levels in brains but increases up to 3-fold during neurodegenerative events, likely in response to the early event of calcium dysregulation, preventing the increase in TGM2 activity by antagonizing CaM-binding offers an option. The therapeutic use of CaM antagonists for the treatment of multiple NDDs has been well-reviewed, but a few updates are important here [100,160]. For example, CaM antagonists have been identified that normalize calcium levels in HD cell lines and decrease TGM2 activity, reducing mHtt cytotoxicity [153,173]. In addition, a novel model for the prediction of CaM-based neuroprotective compounds based on the NIFPTML (Network Invariants, Information Fusion, Perturbation Theory, Machine Learning) models has yielded success with derivatives of Riluzole, a CaM antagonist [174]. Riluzole is approved by the U.S. Food and Drug Administration (FDA) for the treatment of ALS and has been shown to be effective in animal models of HD, PD and others in part due to its strong anticonvulsant, antidepressant, neuroprotective and sedative properties [175]. Continued artificial intelligence-based modeling will guide the development of more precisely targeted, neuro-specific drugs to antagonize CaM and other critical molecules involved in neurodegenerative events.

To gain further understanding of the potential interplay between TGM2, the toxic proteins it aggregates, and CaM, the interactions between CaM and those toxic proteins require analysis. In addition, what is the exact expression level of CaM in different brain regions in different NDDs and its relationship to the activity of TGM2 in these areas?

While this review has focused on a select group of toxic proteins that are found in primary and mixed NDDs and that undergo TGM2-mediated aggregation, TGM2 also causes the aggregation of Prp and neurofilament proteins [28,176]. This suggests that targeting CaM-regulated TGM2 could be a more universal therapeutic approach than discussed here.

## Data Availability

Not applicable.

## Abbreviations

Aβ	amyloid beta
AD	Alzheimer’s disease
ALS	amyotrophic lateral sclerosis
αSyn	alpha-synuclein
Ca^2+^	calcium cations
CaM	calmodulin
CaMBD	calmodulin binding domain
CaMBP	calmodulin-binding protein
FTD	frontotemporal dementia
FUS	fused in sarcoma
HD	Huntington’s disease
Htt	huntingtin
LB	Lewy Body
LBD	Lewy Body Dementia
mHtt	mutant huntingtin
MS	multiple sclerosis
NFTs	neurofibrillary tangles
PD	Parkinson’s disease
PrP	prion protein,
pTau	phosphorylated Tau
ROS	reactive oxygen species
SOD1	superoxide dismutase 1
TDP-43	TAR DNA-binding protein 43
TGM2	transglutaminase 2
